# Porous Poly(Hexamethylene Biguanide) Hydrochloride Loaded Silk Fibroin Sponges with Antibacterial Function

**DOI:** 10.3390/ma13020285

**Published:** 2020-01-08

**Authors:** Ahui Liang, Min Zhang, Hong Luo, Longxing Niu, Yanfei Feng, Mingzhong Li

**Affiliations:** National Engineering Laboratory for Modern Silk, College of Textile and Clothing Engineering, Soochow University, Suzhou 215123, China; lah892778661@163.com (A.L.); zhangmin29usher@163.com (M.Z.); lh824343687@163.com (H.L.); niu18860902006@163.com (L.N.); fengyanfei0123@126.com (Y.F.)

**Keywords:** silk fibroin, poly(hexamethylene biguanide) hydrochloride, sponges, antibacterial materials

## Abstract

In order to endue silk fibroin (SF) sponges with antibacterial function, positively charged poly(hexamethylene biguanide) hydrochloride (PHMB) was incorporated in SF through electrostatic interaction and by freeze-drying technique. The influence of PHMB on the structure and antibacterial activities of SF sponges was investigated. The zeta potential of SF was increased significantly when PHMB was incorporated in SF. The pores with size from 80 to 300 µm and the microscale holes in the pore walls within PHMB-loaded SF sponges provided the channels of PHMB release. The PHMB loaded in the porous sponges showed continuous and slow release for up to 20 days. Effective growth inhibition of both *Escherichia coli* and *Staphylococcus aureus* was achieved when the mass ratio of PHMB/SF was higher than 2/100. These results suggest that the porous PHMB/SF sponges have the potential to be used as a novel wound dressing for open skin wounds.

## 1. Introduction

As temporary wound coverings, wound dressings function as a skin barrier. Wound dressings can not only protect the wound from invasion by external microorganisms but also prevent the evaporation and loss of water and body fluid from the wound, providing a favorable environment for wound healing [1]. In an open wound, skin defects are always accompanied by bacterial infection [2,3]. The necrotic tissue rich in denaturing proteins and moist environment created by wound exudate provide favorable conditions for bacterial growth and reproduction. If bacterial infection is not effectively controlled, the continuous production of bacterial endotoxins and inflammatory factors that stimulate the production of various cells will seriously hinder the healing of the wound, affect the growth rate of the epidermis, or even cause multiple organ failure, eventually threatening the life of patients [4]. Therefore, inhibiting the growth of bacteria at the wound is the key to wound healing [5,6,7].

Silk fibroin (SF), a natural protein with an isoelectric point of approximately 4.2 [8], has a negative charge under neutral conditions. SF has been shown to be promising as a wound dressing due to its excellent biocompatibility, biodegradability, and low immunogenicity [9,10]. When SF dressings cover a skin defect, the NF-kB signaling pathway can be stimulated to control the expression of vimentin, cyclin D1, vascular endothelial cell growth factor, and fibronectin [11,12,13,14], which regulate cell adhesion and proliferation [15] and enhance c-Jun protein expression and phosphorylation to promote wound healing [16]. The results of our previous studies have shown that three-dimensional SF scaffolds prepared by freeze-drying can guide the growth of capillaries, microvessels, and fibroblasts and promote the regeneration of dermal tissue [17,18,19]. However, none of the investigated SF materials showed effective antibacterial function when applied to wound healing.

**Figure 1:**
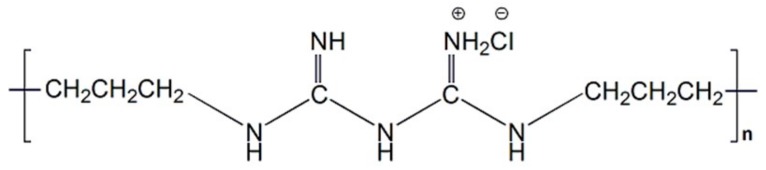
Chemical structure of poly(hexamethylene biguanide) hydrochloride (PHMB).

Poly(hexamethylene biguanide) hydrochloride (PHMB), as a highly effective antimicrobial, is a cationic oligomer with an average of 7–11 biguanide groups spaced by flexible hexamethylene segments, as shown in Figure 1. The mechanism of the antibacterial action of PHMB on the bacterial cytoplasmic membrane has been confirmed: The adhesion of PHMB to the cytoplasmic membrane of bacterial cells causes the cells to leak potassium ions and other components from the cytoplasmic fluid, resulting in cell death [20]. This agent differs from other known antiseptics in that it significantly promotes wound healing [21,22], and it has been widely used in wound infection due to its chemical stability, low cytotoxicity, high effectiveness against microorganisms, and reasonable cost [23,24,25,26].

The aim of this study was to integrate positively charged PHMB into SF through electrostatic interaction and to fabricate porous SF sponges with antibacterial function by freeze-drying technique without the use of a chemical cross-linking agent. The release characteristics of PHMB from the sponges and the antibacterial effects of PHMB on *Staphylococcus aureus* (*S. aureus*) and *Escherichia coli* (*E. coli*) were investigated.

## 2. Materials and Methods

### 2.1. Preparation of Silk Fibroin (SF) Solution

*Bombyx mori* raw silks were degummed following a conventional procedure described previously [27]. Briefly, silk fibers (Huzhou, China) were degummed three times in 0.05 wt % Na_2_CO_3_ aqueous solution at 100 °C for 30 min and dried at 60 °C after rinsing thoroughly with deionized water. The extracted fibers were dissolved in 9.3 M LiBr solution at 60 °C for 1 h. Regenerated SF solution was obtained after dialysis (MWCO, 8–14 kDa) in deionized water for 3 days.

### 2.2. Preparation of Poly(Hexamethylene Biguanide) Hydrochloride (PHMB)/SF Sponges

Glycerin (Sigma-Aldrich, St. Louis, MO, USA) solution was first added to the SF solution at room temperature and stirred for 30 min to form a homogeneous solution with a final SF concentration of 2 wt %. Then, PHMB (Lonza Co., Ltd, Allendale, NJ, USA) solution was added to the mixture to make blend solutions with final PHMB/SF mass ratios of 0/100, 0.5/100, 1/100, 2/100, 5/100, and 10/100, respectively. After removing the bubbles inside the solution, the mixture was cast onto a stainless steel plate in a layer with a constant thickness of approximately 2 mm. The plates were placed in a freezing chamber for 6 h at −40 °C, followed by freeze-drying for 36 h to form the porous PHMB/SF sponges. Then, the sponges were placed in a constant temperature and humidity incubator and treated at 40 °C and 90% relative humidity for 24 h, making the porous sponges insoluble in water. Finally, the sponges were sterilized with γ-ray irradiation and stored at 4 °C before use.

### 2.3. Zeta Potential of the PHMB/SF Complexes

The zeta potentials of the PHMB/SF complexes were measured with a Zetasizer Nano ZS90 (Malvern, UK) at 25 °C. The complexes were prepared at PHMB/SF mass ratios of 0/100, 0.5/100, 1/100, 2/100, 5/100, and 10/100 according to the procedures described above. The blended solutions were diluted with distilled water to 1 mL for zeta potential measurements. Each sample was run for 300 seconds and analyzed in a unimodal analysis mode. Measurements for each sample were repeated three times.

### 2.4. Scanning Electron Microscopy (SEM)

The cross-sectional morphology of the porous PHMB/SF sponges was observed using a scanning electron microscope (SEM; S-4800, Hitachi, Tokyo, Japan). Samples were mounted on a copper plate and sputter-coated with a gold layer of 20–30 nm thickness prior to imaging.

### 2.5. Fourier Transform Infrared (FTIR) Spectroscopy

The porous PHMB/SF sponges were cut into microparticles with radii less than 40 µm, and samples were prepared in KBr pellets. Fourier transform infrared (FTIR) spectra were obtained with a Nicolet 5700 (Thermo Fisher Scientific Inc, Waltham, MA, USA).

### 2.6. PHMB release from the PHMB/SF Sponges

The assay was conducted according to Bueno’s route with minor modifications [28]. The release characteristics of PHMB from the porous PHMB/SF sponges were measured by immersing the sponges (20.0 × 20.0 × 2.0 mm^3^) in 10 mL of phosphate buffer saline (PBS, 10 mM, pH = 7.4) in centrifuge tubes, which were kept in a shaking incubator with a shaking speed 60 rpm at 37 °C. At specific time intervals, 2.0 mL of solution was withdrawn, and equal amounts of fresh PBS were replaced. The drug concentration was evaluated by UV spectroscopy using a Smart-Spec TM Plus Spectrometer (Bio-Rad, Hercules, CA, USA). Calibration curves were obtained by plotting the absorbance measured at 235 and 276 nm versus the PHMB and SF concentrations, respectively. All release tests were carried out using three replicates and the results were averaged. 

### 2.7. Antibacterial Activity Test

The antibacterial activity tests against *Staphylococcus aureus* (ATCC 25923, Manassas, WA, USA) and *Escherichia coli* (ATCC 25922, Manassas, WA, USA) were carried out using the agar disk diffusion method described by Schillinger and Lücke [29]. The samples were cut into circular pieces (10.0 mm diameter, 2.0 mm thickness). A control sample was prepared by preparing the porous SF sponges without PHMB. All samples were sterilized by irradiation with ^60^Co. The plates were filled with agar medium. A transfer loop of bacteria was taken and evenly dispersed on the surface of the agar medium. After the plate was inoculated, the samples were placed on the plate and placed in the incubator at 37 °C for 24 h.

### 2.8. Statistical Analysis

All data are expressed as the mean ± standard deviation (SD). Statistical comparisons were performed using one-way analysis of variance (t-test), and differences at *P* < 0.05 were considered statistically significant.

## 3. Results

### 3.1. Zeta Potential of the PHMB/SF Complexes

**Figure 2:**
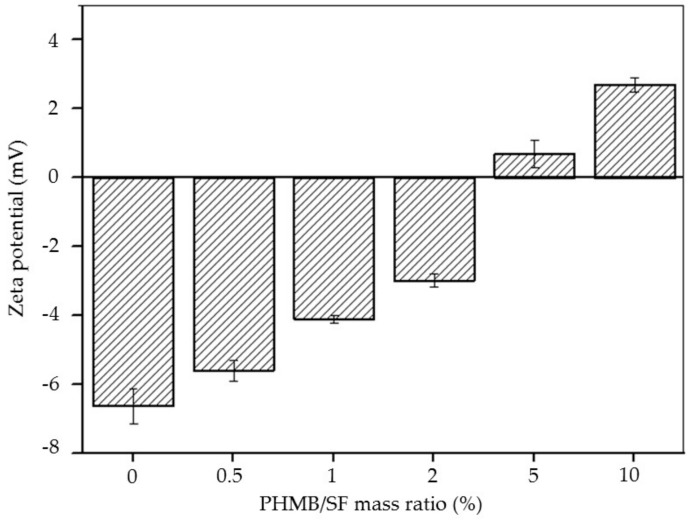
Zeta potential of poly(hexamethylene biguanide) hydrochloride (PHMB)/silk fibroin (SF) complexes at different w/w ratios.

As shown in Figure 2, the zeta potential of SF was −6.64 ± 0.53 mV. As the mass ratio of PHMB/SF increased, the zeta potential of the complexes increased. The zeta potential of the complexes was +0.68 ± 0.40 mV when the mass ratio of PHMB/SF reached 5/100, indicating that the negative charge on the surface of SF protein was completely neutralized and reversed from negative to positive. The zeta potential of the complexes increased to +2.70 ± 0.26 mV when the mass ratio of PHMB/SF was 10/100, which showed that the surface of complexes had a large positive charge.

### 3.2. Morphology of PHMB/SF Sponges

**Figure 3:**
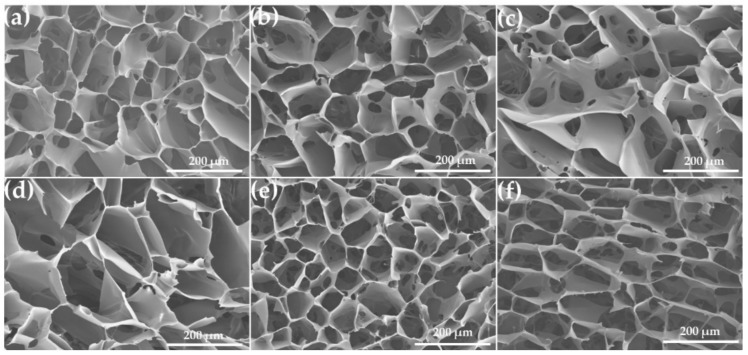
Scanning electron microscopy (SEM) images of the cross-section for porous PHMB/SF sponges. PHMB/SF ratios: (**a**) 0/100; (**b**) 0.5/100; (**c**) 1/100; (**d**) 2/100; (**e**) 5/100; (**f**) 10/100.

The microstructure of the inner pores of PHMB/SF sponges with different blend ratios is shown in Figure 3. The shape of the pores in pure SF sponges presented an irregular polygon or ellipse with diameters ranging from 80 to 300 µm (Figure 3a). There were some microscale holes with a size of 5–40 µm in the pore walls within the sponges (Figure 3a–f). No obvious morphological changes and phase separation were observed in the sponges after incorporating PHMB in SF with PHMB/SF ratios of 0.5/100, 1/100, and 2/100 (Figure 3b–d). When the mass ratio of PHMB/SF reached 5/100 (Figure 3e) and 10/100 (Figure 3f), more microscale holes appeared in the walls of pores, and the connectivity between pores was increased. The pores with size from 80 to 300 µm and the microscale holes in the pore walls within PHMB loaded SF sponges provided the channels of PHMB release.

### 3.3. FTIR Spectra

Studies on the molecular conformation of SF have shown that SF usually shows characteristic absorption bands at approximately 1650–1655 cm^−1^ (amide I), 1525–1540 cm^−1^ (amide II), 1266 cm^−1^ (amide III), and 669 cm^−1^ (amide V), representing α-form structure; bands at approximately 1620–1635 cm^−1^ (amide I), 1531 cm^−1^ (amide II), 1230–1235 cm^−1^ (amide III), and 695 cm^−1^ (amide V), attributed to β-sheet; and bands at 1655–1660 cm^−1^ (amide I), 1535–1545 cm^−1^ (amide II), 1235 cm^−1^ (amide III), and 650 cm^−1^ (amide V), assigned to random coil conformation [30,31].

**Figure 4:**
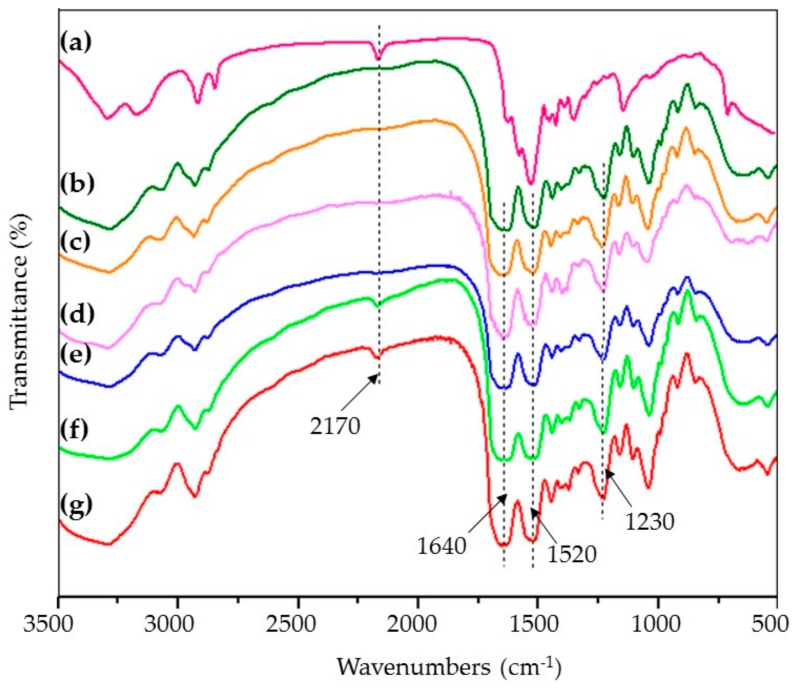
Fourier transform infrared (FTIR) spectra of porous PHMB/SF sponges. PHMB/SF ratios: (**a**) 100/0; (**b**) 0/100; (**c**) 0.5/100; (**d**) 1/100; (**e**) 2/100; (**f**) 5/100; (**g**) 10/100.

The spectrum of the pure SF sponges showed strong absorption bands at approximately 1640 cm^−1^, 1520 cm^−1^, and 1230 cm^−1^, indicating the presence of a β-sheet conformation in the porous SF sponges (Figure 4b). The spectrum of the PHMB (Figure 4a) showed an absorption band at 2170 cm^−1^, which was attributed to C=N stretching vibrations [32]. As the loading content of PHMB increased in the porous PHMB/SF sponges, the characteristic absorption of SF did not change obviously. When the mass ratio of PHMB/SF was 5/100 (Figure 4f) and 10/100 (Figure 4g), the characteristic peak of C=N at 2170 cm^−1^ appeared, which indicated that PHMB was effectively loaded into the porous SF sponges. 

### 3.4. Release of PHMB from the PHMB/SF Sponges

**Figure 5:**
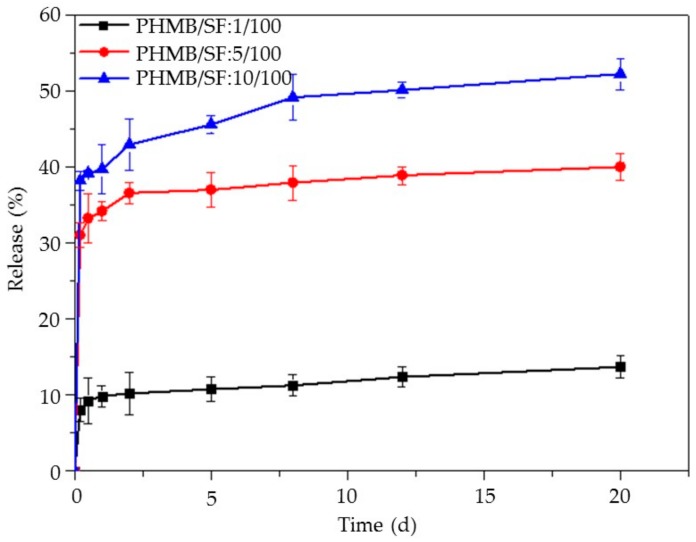
Release of PHMB from PHMB/SF sponges upon immersing the sponges in PBS at 37 °C. PHMB/SF ratios: 1/100, 5/100, 10/100.

The release of PHMB from the porous PHMB/SF sponges with the PHMB/SF ratios of 1/100, 5/100, and 10/100, respectively, in 20 days is shown in Figure 5. The release profiles of all test samples showed that the PHMB loaded in the porous sponges could slowly release for more than 20 days. An initial burst release of PHMB was observed in the first 12 h, followed by steady release for the next period in all test samples. The cumulative release of PHMB during 20 days was 12.8% when the mass ratio of PHMB/SF was 1/100. With an increase in PHMB loading, the cumulative release increased. When the mass ratio of PHMB/SF reached 5/100 and 10/100, the cumulative release of PHMB was 39.8% and 53.5%, respectively, which illustrated that the sponges with high PHMB loading had a greater release of PHMB. The gradual release of PHMB from the porous SF sponges over the course of 20 days might endue the sponges with the potential to continuously inhibit bacterial proliferation.

### 3.5. Antibacterial Activity Test

**Figure 6:**
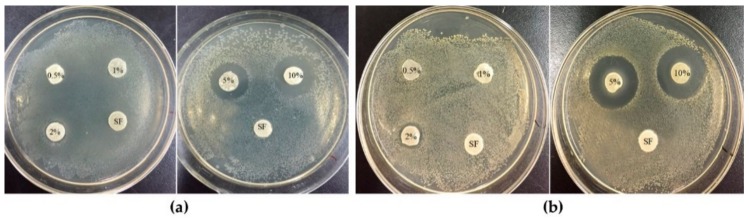
Bacterial growth inhibition by the disk method. Porous PHMB/SF sponges were incubated at 37 °C for 24 h on agar plates cultured with (**a**) *E. coli* and (**b**) *S. aureus*. 0.5%, 1%, 2%, 5%, and 10% represent the sponges with PHMB/SF ratios of 0.5/100, 1/100, 2/100, 5/100, and 10/100, respectively. SF represents PHMB free sponges.

The antibacterial activity of the porous PHMB/SF sponges was investigated by the disk diffusion method against *E. coli* and *S. aureus*, as shown in Figure 6. When the test disks were applied onto the surface of inoculated agar medium, the wetted disks absorbed water from the test medium, the release of the antimicrobial agent was initiated, and PHMB migrated through the adjacent agar medium. As a result, a gradually changing concentration gradient of the antibacterial agent developed in the agar surrounding the disk. As shown in Figure 6a,b, no clear inhibition zone was observed when the mass ratios of PHMB/SF were 0.5/100 and 1/100, and the same results were observed for the PHMB free SF sponges. However, when the mass ratio of PHMB/SF was 2/100, the samples began to show obvious inhibition zones of 10.5 mm and 10.67 mm for *E. coli* (Figure 6a) and *S. aureus* (Figure 6b), respectively. With an increase in the PHMB loading in the porous PHMB/SF sponges, the inhibition effect increased. When the mass ratio of PHMB/SF reached 10/100, the inhibition zone increased to 15.8 mm (Figure 6a) and 18.6 mm (Figure 6b) for *E. coli* and *S. aureus*, respectively. Hence, the porous PHMB/SF sponges exhibited effective antibacterial activity when the mass ratio of PHMB/SF was higher than 2/100.

## 4. Discussion

It is difficult for systemically applied antibiotics to reach wounds because of poor blood circulation. The application of local antibiotics is the main method used to prevent wound infection [33]. The effective strategy is to load the antibacterial drugs into the wound dressing and release it slowly to the wound to achieve a long-lasting local antibacterial effect. An ideal wound dressing should: 1) Be close to the wound to absorb excess exudate and maintain a moist environment in the wound; 2) be nontoxic and have no immunogenicity; and 3) maintain moisture absorption ability, block the invasion of external bacteria and pathogenic organisms, and be able to release antibacterial drugs slowly to prevent infection of the wound [34,35,36]. In this study, we fabricated porous PHMB/SF sponges with antibacterial function through electrostatic interaction and by freeze-drying. 

Positively charged PHMB was combined with negatively charged SF mainly through electrostatic interaction. The results showed that the zeta potential of the PHMB/SF complex was reversed from −6.64 ± 0.53 mV to +2.70 ± 0.26 mV when the mass ratio of PHMB/SF reached 10/100 (Figure 2) because of the adsorption of PHMB on SF protein surface and the neutralization with the negative charge on SF. The prepared PHMB/SF sponges (Figure 3a–f) had relatively uniform pores with diameters between 80 and 300 µm. When the mass ratios of PHMB/SF were 5/100 and 10/100 (Figure 3e,f), more microscale holes with size of 5–40 µm in the pore walls appeared, and the connectivity between pores was significantly improved. The interconnected porous structure was conductive to the release of PHMB from the porous sponges to achieve antibacterial effects.

The FTIR results (Figure 4c–g) indicated that the molecular conformation of SF in the porous PHMB/SF sponges was not significantly affected by the incorporation of PHMB, and was mainly characterized by a β-sheet conformation due to the addition of glycerol [37]. The absorption bands of the C=N stretching vibrations in PHMB/SF sponges (Figure 4f,g) appeared at 2170 cm^-1^, which was the characteristic absorption of loaded PHMB (Figure 4a).

When the PHMB was added into SF solution, the positively charged PHMB was combined with negatively charged SF by electrostatic interaction, hydrogen bonding, and Vander Waals’ force, dispersed in continuous SF phase, and was embedded by SF networks in porous SF sponges after freeze-drying. The release experiment was designed to investigate the release characteristics of loaded PHMB from the sponges. The release profiles showed that the PHMB was burst released in the first 12 h and followed by steady release for the next period from the SF sponges for over 20 days (Figure 5). The loaded PHMB experienced an initial burst release because of the weak physical forces between SF and PHMB, such as Vander Waals’ force, hydrogen bonding, and electrostatic adsorption, which were easily broken [38]. The reason for subsequent slow release could be considered to be that the PHMB needed to surmount not only the weak physical forces between SF and PHMB, but also the diffusion barriers created by surrounding SF networks. It may be an effective way to increase the cumulative release of PHMB from the porous sponges by reducing the concentration of SF to improve the porosity of the sponges and decrease the SF barrier. Compared with PHMB-loaded chitosan/poly(ethylene oxide) electrospun membranes exhibiting a quick release in the 24 h with approximately 85% of PHMB released from the nanofibers [39], or PHMB-loaded PLA scaffolds showing a quick release pattern during 7 h [40], the loaded PHMB in porous SF sponges in this study could continuously and slowly release for up to 20 days, and the burst release of PHMB could effectively inhibit the bacterial proliferation in the early stage of wound healing. 

PHMB has been used as an antibacterial agent for many years with proven effectiveness against a broad number of bacterial and fungal species with rapid and sustained action [23,24,25,26]. As shown in Figure 6a,b, the porous PHMB/SF sponges had an obvious inhibitory effect on the growth of *E. coli* and *S. aureus* when the mass ratio of PHMB/SF was over 2/100. With increased PHMB loading in the PHMB/SF sponges, the inhibition effect increased, which indicated that the antibacterial activity of the PHMB/SF sponges was dependent on the drug concentration. Loading too much PHMB into skin scaffolds might cause cytotoxicity [41]. What proportion of PHMB is suitable for loading in the SF sponges remains to be further studied. Compared with antimicrobial peptide-immobilized silk fibroin membrane using EDC/NHS crosslinking [42], the sponges in this study avoided the use of chemical crosslinking agent and could also achieve effective antibacterial activity. Those results proved that porous PHMB/SF sponges have the potential to be used as a novel wound dressing to effectively inhibit bacterial proliferation during wound healing. 

## 5. Conclusions

A novel porous SF sponge with antibacterial function through incorporating positively charged PHMB to negatively charged SF was fabricated by freeze-drying. The zeta potential of the PHMB/SF complexes increased significantly when PHMB was incorporated in SF protein. When the mass ratio of PHMB/SF was higher than 5/100, the zeta potential of the complex reversed from negative to positive. SEM images showed that the pore size of the porous sponges ranged from 80 to 300 µm, which provided channels for PHMB release. The release profiles showed that PHMB loaded in porous SF sponges could slowly release for more than 20 days. Antibacterial tests showed that the porous sponges had a significant inhibitory effect on *E. coli* and *S. aureus* when the mass ratio of PHMB/SF was higher than 2/100. The porous PHMB/SF sponges have the potential to be a novel wound dressing for covering open skin wounds.
